# Analyses of Experimental Dental Adhesives Based on Zirconia/Silver Phosphate Nanoparticles

**DOI:** 10.3390/polym15122614

**Published:** 2023-06-08

**Authors:** Abdul Samad Khan, Yasmin Alhamdan, Hala Alibrahim, Khalid S. Almulhim, Muhammad Nawaz, Syed Zubairuddin Ahmed, Khalid Aljuaid, Ijlal Shahrukh Ateeq, Sultan Akhtar, Mohammad Azam Ansari, Intisar Ahmad Siddiqui

**Affiliations:** 1 Department of Restorative Dental Science, College of Dentistry, Imam Abdulrahman Bin Faisal University, Dammam 34212, Saudi Arabia; ksalmulhim@iau.edu.sa (K.S.A.); szahmed@iau.edu.sa (S.Z.A.); 2 College of Dentistry, Imam Abdulrahman Bin Faisal University, Dammam 34212, Saudi Arabia; yasminalhamdan15@gmail.com (Y.A.); 2230600011@iau.edu.sa (H.A.);; 3 Department of Nano-Medicine Research, Institute for Research and Medical Consultations (IRMC), Imam Abdulrahman Bin Faisal University, Dammam 31441, Saudi Arabia; mnnmuhammad@iau.edu.sa; 4 Department of Biomedical Engineering, College of Engineering, Imam Abdulrahman Bin Faisal University, Dammam 34212, Saudi Arabia; 5 Department of Biophysics, Institute for Research and Medical Consultations (IRMC), Imam Abdulrahman Bin Faisal University, Dammam 31441, Saudi Arabia; 6 Department of Epidemic Disease Research, Institutes for Research and Medical Consultations (IRMC), Imam Abdulrahman Bin Faisal University, Dammam 31441, Saudi Arabia; maansari@iau.edu.sa; 7 Department of Dental Education, College of Dentistry, Imam Abdulrahman Bin Faisal University, Dammam 34212, Saudi Arabia

**Keywords:** dental adhesives, adhesion, zirconia, silver, phosphate, nanoparticles, mechanical properties, degree of conversion, bond strength, color stability

## Abstract

This study aimed to evaluate the incorporation of zirconia/silver phosphate nanoparticles to develop experimental dental adhesives and to measure their physical and mechanical properties. The nanoparticles were synthesized by the sonication method, and the phase purity, morphological pattern, and antibacterial properties with *Staphylococcus aureus* and *Pseudomonas aeruginosa* were assessed. The silanized nanoparticles were incorporated (0, 0.15, 0.25, and 0.5 wt.%) into the photoactivated dimethacrylate resins. The degree of conversion (DC) was assessed, followed by the micro-hardness and flexural strength/modulus test. Long-term color stability was investigated. The bond strength with the dentin surface was conducted on days 1 and 30. The transmission electron microscopy and X-ray diffractogram confirmed the nano-structure and phase purity of the particles. The nanoparticles showed antibacterial activities against both strains and inhibited biofilm formation. The DC range of the experimental groups was 55–66%. The micro-hardness and flexural strength increased with the concentration of nanoparticles in the resin. The 0.5 wt.% group showed significantly high micro-hardness values, whereas a non-significant difference was observed between the experimental groups for flexural strength. The bond strength was higher on day 1 than on day 30, and a significant difference was observed between the two periods. At day 30, the 0.5 wt.% showed significantly higher values compared to other groups. Long-term color stability was observed for all the samples. The experimental adhesives showed promising results and potential to be used for clinical applications. However, further investigations such as antibacterial, penetration depth, and cytocompatibility are required.

## 1. Introduction

Since the 1950s, polymer-based dental adhesives have been extensively used and improvised, and new materials have emerged in the last decade due to the increased demand of clinicians and patients [1]. Despite the continuous improvements in adhesive systems, resin restoration manifests a weak spot at the bonded interface [2]. A failure rate of 15–50% of resin-based composite restorations has been documented [3]. Failure in the resin–dentin bonding interface can lead to secondary caries, marginal deficiency, staining, post-operative sensitivity, and micro-movement between the materials [4,5].

Adhesives can be modified by reinforcing the adhesive layer and performing a specific function with improved chemical stability and mechanical and physical properties [6,7]. Improvement in properties is dependent on the concentration of the fillers. The higher loading of fillers can negatively affect the bond strength, penetration of adhesives in dentinal tubules, and the polymerization rate. Adding an antibacterial agent can minimize biofilms and bacterial ingress, subsequently reducing the recurrence of secondary caries at the tooth margins [8]. Imazato et al. introduced the “immobilized bactericide” concept to inhibit bacteria that come into contact with dental resin materials [9]. With the emergence of nanotechnology in dentistry, nanoparticles of different origins such as silica [10], zirconia [11], amorphous calcium phosphate [12], hydroxyapatite [13], titanium dioxide [14], zinc oxide [15], nano-clay [16], silver [17], etc. have been added to improve the mechanical and antibacterial properties of the dental adhesives.

Silver (Ag), a potent antibacterial metal ion nanoparticle, exhibits a high surface area when used in a low filler concentration in the adhesive. Furthermore, Ag-based adhesives can deliver a high antibacterial effect without influencing the restoration’s shade and mechanical properties [18]. It is reported that Ag_3_PO_4_ (silver orthophosphate) effectively killed bacteria and fungi [19,20,21,22]. The antibacterial activity of Ag_3_PO_4_ is mostly enhanced after incorporating it with other nanoparticles [23,24,25]. Zirconium oxide (ZrO_2_) provides high strength, high toughness, high corrosion resistance, low toxicity, biocompatibility, and antibacterial properties [26]. Incorporating ZrO_2_ in different materials/matrices can improve fracture toughness, flexural strength, shear bond strength, and optical properties [27,28].

The bond strength of adhesion to dentin relies mainly on the hybrid layer, and many attempts have been conducted to improve the mechanical properties of this hybrid layer [29]. Forming hydrolytically stable calcium salts “nano-layering” at the resin adhesive interface can increase the bond strength [30]. Combining different ions to enhance the adhesive systems is advocated due to the great challenge of having one agent with various desirable properties. In addition, the combined effects from other agents can improve the resin–dentin bond durability. The current data in the literature regarding the antibacterial efficacy and the physical and mechanical properties of these nanoparticles are sparse. Thus, this study aims to evaluate whether the addition of ZrO_2_/Ag_3_PO_4_ nanoparticles into a dental resin adhesive would affect its physical and mechanical properties. The nanoparticles were synthesized, and their structural, morphological, and antibacterial properties were investigated. After adding the nanoparticles in dental resins, the degree of conversion, micro-hardness, flexural strength, and flexural modulus were analyzed. The bond strength and color stability were analyzed at different time intervals. It was hypothesized that the addition of combined nanoparticles could enhance the mechanical properties of the dental resin adhesives and that the color of ZrO_2_/Ag_3_PO_4_ would not affect the restorations’ optical properties.

## 2. Materials and Methods

### 2.1. Preparation of Nanoparticles

All chemicals used in this study were of analytical grade and purchased from Sigma Aldrich, St. Louis, MO, USA. Initially, 0.6 g zirconyl chloride was dissolved in 25 mL of ethylene glycol and continuously stirred. Afterward, 0.3 g sodium hydroxide and 0.2 g polyvinylpyrrolidone (PVP) were added to the solution and sonicated for 40 min. The product was centrifuged, washed with water and ethanol, and dried overnight. Then, to prepare ZrO_2_/Ag_3_PO_4_ nanoparticles, 0.3 g ZrO_2_ was dispersed in 30 mL of water; then, 0.5 g silver nitrate was added to it with continued stirring. Then, 0.3 g disodium hydrogen phosphate (Na_2_HPO_4_) in 10 mL water was added dropwise to the above solution and stirred for 5 min more. Next, the mixture was sonicated for 1 h, centrifuged, washed, and dried.

### 2.2. Synthesis of Experimental Dental Adhesive

All chemicals used for resin-based adhesives were purchased from Sigma Aldrich, St. Louis, MO, USA. The matrices in this study were high molecular weight monomers, i.e., bisphenol A glycol dimethacrylate (bis-GMA), urethane dimethacrylate (UDMA), and low molecular weight monomers, i.e., triethylglycol methacrylate (TEGDMA). The dimethacrylate resin (bis-GMA: UDMA; TEGDMA) ratio was optimized to 40:35:25 with 20% ethanol (m/m). The combined concentration of the photoinitiating system, i.e., camphorquinone (CQ) and ethyl 4-dimethylaminobenzoate (EDBA), was 1 wt.%. The resinous monomers were mixed at room temperature with a digital overhead stirrer for 1 h; then, CQ (0.5 wt.%) and EDAB (0.5 wt.%) were mixed in resins and allowed to stir for 30 min. The procedure was performed in a dark light to avoid premature polymerization. The unfilled resin was labeled as Exp-0. Universal adhesive Tetric N-bond universal (TEU, Ivoclar Vivadent; Schaan, Liechtenstein) was used as a negative control material (Com).

The prepared nanoparticles were silanized with 1.0 vol.% γ-methacryloxypropyltrimethoxy silane (MPS, Sigma Aldrich, St. Louis, MO, USA) as described previously [15]. The silanized nanoparticles were mixed with each combination of prepared resins. The nanoparticle concentrations were 0.15 wt.%, 0.25 wt.%, and 0.5 wt.%. The incremental addition of nanoparticles was conducted to allow uniform distribution of fillers in resin. Furthermore, the ultrasonic probe sonicator (QSONICA sonicator, Newtown, CT, USA) was used to get uniform distribution of nanoparticles. The obtained experimental adhesives were packed in airtight and dark vials for further analysis. The samples were labeled Exp-0.15, Exp-0.25, and Exp-0.5 based on the concentration of the nanoparticles.

### 2.3. Characterizations

The phases and crystal structure of ZrO_2_/Ag_3_PO_4_ nanoparticles were determined by X-ray diffraction (XRD, Rigaku, Tokyo, Japan). SEM (TESCAN, Brno, Czech Republic) equipped with an energy-dispersive X-ray spectrometer (EDX) was utilized to study the elemental composition of ZrO_2_/Ag_3_PO_4_. The morphology and size of nanoparticles were determined by field emission electron microscope (FE-TEM, JEOL, Tokyo, Japan). UV-visible diffuse reflectance spectrum (DRS) was obtained on a UV-visible spectrophotometer (JASCO-V-750, Helsinki, Finland).

#### 2.3.1. Antimicrobial and Anti-Biofilm Assessment

Multidrug-resistant *Pseudomonas aeruginosa* (MDR-PA) and methicillin-resistant *Staphylococcus aureus* (MRSA) were used to assess the antibacterial and anti-biofilm activity of ZrO_2_/Ag_3_PO_4_.

##### Minimal Inhibitory Concentration (MIC) and Minimal Bactericidal Concentration (MBC)

The MIC and MBC values of ZrO_2_/Ag_3_PO_4_ against the MDR-PA and MRSA were assessed by using the micro-broth dilution procedure described elsewhere [22]. In brief, 20 µL of freshly grown cultures (≈1 × 10^6^ cfu/mL) were suspended in 180 µL of BHI broth containing varying concentrations (0.125 to 16 mg/mL) of ZrO_2_/Ag_3_PO_4_ for 24 h at 37 °C.

##### Inhibition of Biofilm Formation

The biofilm formation inhibition after treatment with ZrO_2_/Ag_3_PO_4_ was quantitatively assessed by crystal violet assay. Briefly, fresh cultures of 20 µL of both bacteria (MRSA and MDR-PA) were inoculated with 180 µL of varying concentrations (31.25 to 500 µg/mL) of ZrO_2_/Ag_3_PO_4_ for 24 h. The cells without ZrO_2_/Ag_3_PO_4_ were taken as control. After 24 h of incubation, the content from each well was decanted and washed gently with PBS, and the well was left for drying. In the next step, the adhered biofilm was stained with crystal violet for 20 min and then washed with PBS, and the wells were dried completely. The stained biofilm was then solubilized with 95% ethyl alcohol, and finally, the optical density was taken at 595 nm to analyze the percent inhibition of biofilm formation [21,22].

#### 2.3.2. Structural and Degree of Conversion Analyses

The structural and degree of conversion (DC) were evaluated using Fourier Transform Infrared Spectroscopy (FTIR; Thermo Fisher Scientific, Waltham, MA, USA). A Teflon mold was placed on the FTIR window and samples were dropped in the mold in an uncured form in a dark environment. Slight air pressure was applied to allow the solvent to evaporate. Then, each sample was covered with a mylar strip and spectral analysis was conducted. Then, the same sample was cured with light-emitting diode (LED) high-intensity blue light (wavelength: 470 nm; light intensity: 1200 MW/cm^2^, Woodpecker LED, Shanghai, China) for 40 s, and spectral analysis was performed again. The spectral resolution was 4 cm^−1^, and the range was 4000–400 cm^−1^. The degree of conversion was calculated from both peak height and peak area analysis using the standard formula:

DC% = 100 × (R*polym*/R*unpolym*) (1)

where R*polym* = ratio of polymerized aliphatic (1642 cm^−1^) and aromatic (1608 cm^−1^) carbon bonds and R*unpolym* = ratio of unpolymerized aliphatic and aromatic carbon bonds.

#### 2.3.3. Micro-Hardness Testing

The Knoop micro-hardness (MicroMet 6040, Lake Bluff, IL, USA) testing was conducted as per ASTM E384-11e1. Five samples of each group were prepared using a 3D-printed silicon mold with the dimension of 6 × 2 mm^2^. The samples were cured as mentioned above and were polished (MetaServTM 250 Grinder-Polisher with Vector Power Head, Buehler, Lake Buff, IL, USA) using sandpaper with increasing grit (#600, 1000, and 2000). Three indents were taken on each sample, whereby the load was 50 g with a dwell time of 15 s.

#### 2.3.4. Flexural Strength & Flexural Modulus

The three-point flexural strength and flexural modulus analyses were conducted per ISO 4049 specifications. The samples (*n* = 10) were prepared in 25 mm × 2 mm × 2 mm Teflon mold and cured for 40 s from both sides. Before curing, slight air pressure was applied to allow the solvent to evaporate and a mylar strip was placed gently on the surface of mold. After polishing, the samples were stored in deionized water for 24 h at 37 °C prior to the experiment. The samples were loaded on a universal testing machine (Instron 8871; Instron, Norwood, MA, USA). The cross-head speed was 0.5 mm.min^−1^ under the static load of 1 kN. The force was applied until the samples were fractured. The flexural strength was calculated using the following equation:

FS = 3 F d/2 wh^2^ (2)

where F = maximum force, d = distance between the two anchors, w = width of the specimen, and h = height of the specimen

The modulus was calculated using the formula:

E_f_ = FL^3^/4 BH^3^d (3)

where F = maximum load; L = distance between the supports; B = width of the specimen, H = height of the specimen, and d = deflexion (in millimeters) corresponding to the load F.

#### 2.3.5. Bond Strength Analysis

After obtaining approval from the institutional review board (IRB 2022-02-156), a total of 64 caries-free human extracted premolars were collected. The teeth were sterilized (70/30 ethanol solution for 15 min) and stored in 0.5 wt.% thymol solution. The teeth were distributed equally among the groups. The dentin surface was exposed after removing the coronal enamel using a tooth–sawing machine (IsoMet 5000, Lake Bluff, IL, USA). Before applying adhesives, the dentin surface was treated with 37% phosphoric acid (FineEtch, Incheon, Republic of Korea) for 10 s (as per manufacturer’s guidelines) and was washed and dried thoroughly. A layer of adhesive was applied with a micro-applicator on the dentin surface and spread with gentle air pressure. The applied adhesive layer was light-cured with high-intensity blue light for 20 s. Then, the 4 mm × 4 mm resin-based composite (Empress Direct, Ivoclar Vivadent; Schaan, Liechtenstein) was placed on the tooth surface in increments (2 mm of each layer). Each incremental layer was cured for 40 s with high-intensity LED blue light. After curing, the samples were stored in deionized water at 37 °C. Shear bond strength testing was conducted on days 1 and 30 using an Instron testing machine (Instron 8871; Instron, Norwood, MA, USA). The knife-shaped jig (1.5 mm tip) was applied at an interface of adhesive/composite, whereby the cross-head speed was 0.5 mm/min.

After de-bonding, dentin surfaces were examined under an optical microscope (10× magnification) to measure the Adhesive Remnant Index (ARI). After determining the ARI, samples from each group were selected to be examined under scanning electron microscopy (SEM; TESCAN, VEG 3, Brno, Czech Republic). In addition, energy dispersive X-ray spectrometry (EDX) was performed to evaluate the elemental presence. The samples were sputtered for gold-coated (Quorum Technologies, Lewes, UK) for 90 s and images were taken at multiple magnification utilizing the voltage of 15 kV.

#### 2.3.6. Color Stability Measurement

To measure the color stability of control and experimental adhesives, a total of 50 human extracted molars were selected. The teeth were prepared and sectioned to expose the dentin surface as mentioned above. The dentin surface was etched for 10 s and washed/dried. The tooth samples were equally divided randomly into each group (*n* = 10). A single layer of control and experimental adhesives was applied using a micro-brush, and gentle air pressure was applied to allow the solvent to evaporate. The adhesive layer was cured with high-intensity blue light for 20 s. A single layer of 1.5 mm of commercial composite (Filtek Z350 XT Universal Restorative, Shade A2, 3M ESPE, Seefeld, Germany) was applied on the surface and cured for 40 s. The surface was polished (MetaServTM 250 Grinder-Polisher with Vector Power Head, Buehler, Lake Buff, IL, USA) using sandpaper with increasing grit (#600, 1000, and 2000). The samples were placed in deionized water for a periodic time interval, i.e., days 0, 30, and 60 and the color stability measurement was analyzed with the Color-Eye 7000A spectrophotometer (X-rite, Grand Rapids, MI, USA) as per the International Commission on Illumination (Commission internationale d’eclairage—CIE) L* a* b* (CEILab) specifications mentioned previously [31]. The color stability (Δ*E*) was calculated using the following formula:

(4) $$\Delta E=\sqrt{{\left(\Delta L*\right)}^{2}+(\Delta {a*)}^{2}+(\Delta {b*)}^{2}}$$

where ΔL*, Δa*, Δb* represent differences in L*, a*, b*, respectively, before and after immersion in deionized water.

### 2.4. Statistical Analysis

The statistical analysis was performed using SPSS version 22 (IBM Software, Armonk, NY, USA). One-way analysis of variance (ANOVA) post hoc Tukey’s test was applied for all analyses, followed by the honest significant difference (HSD) test, and repeated measurement analysis was also conducted for color stability. The *p*-value of 0.05 was considered significant.

## 3. Results

### 3.1. Nanoparticle Analyses

**Figure 1:**
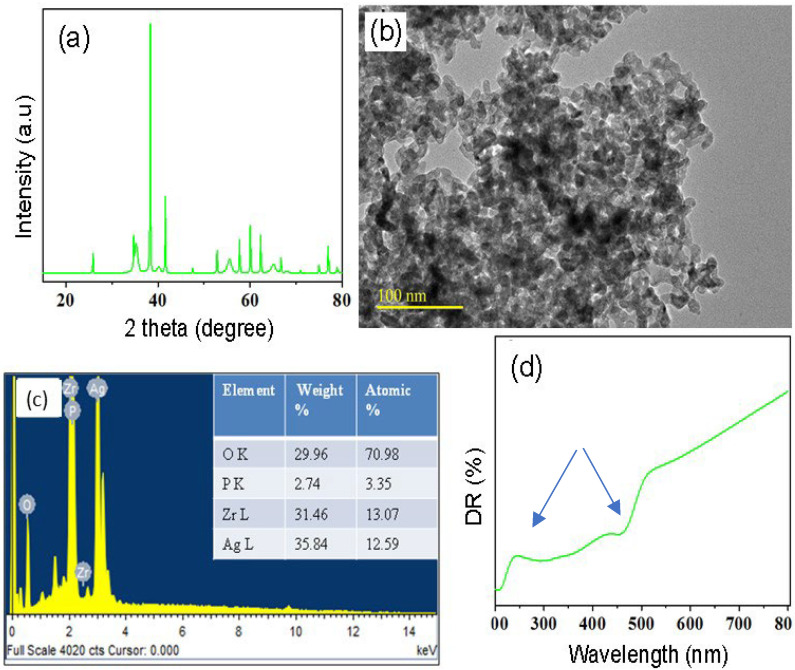
Structural, morphological, and optical characterization of the prepared nanocomposite. (**a**) XRD pattern, (**b**) TEM image, (**c**) EDX analysis, and (**d**) DR-UV spectra of ZrO_2_/Ag_3_PO_4_ nanoparticles where the arrows show the absorption range at 245 nm and 450 nm.

The XRD pattern of ZrO_2_/Ag_3_PO_4_ nanoparticles is presented in Figure 1a, indicating the crystalline nature of ZrO_2_/Ag_3_PO_4_ nanoparticles. It further demonstrated the purity of the nanoparticles. The morphology and size of the nanoparticles were investigated by TEM (Figure 1b) and showed the formation of small particles with an average range size 20–100 nm. Furthermore, EDX analysis (Figure 1c) was performed to know the composition of the prepared nanoparticles and it indicated the presence of Zr, O, Ag, and P. DR-UV-visible spectra of ZrO_2_/Ag_3_PO_4_ nanoparticles were also recorded in the range 200–800 nm, and it showed that nanoparticles exhibited absorption in the visible range (Figure 1d). The optical band gap of nanoparticles was observed around 2.75 eV, whereby the absorption was observed at 245 nm and 450 nm. The increased absorption of zirconium oxide is ascribed due to addition/incorporation of Ag_3_PO_4_ nanoparticles.

### 3.2. Antimicrobial Assessment

**Figure 2:**
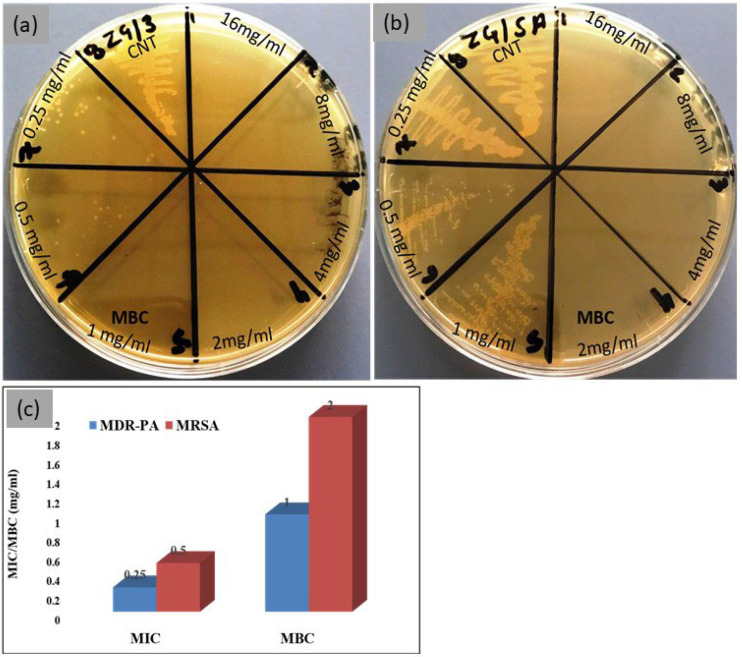
Plates showing MBC values of ZrO_2_/Ag_3_PO_4_ against (**a**) MDR-PA and (**b**) MRSA. (**c**) Showing MIC and MBC (mg/mL).

The MICs values of ZrO_2_/Ag_3_PO_4_ against MDR-PA and MRSA were 0.25 mg/mL and 0.5 mg/mL, respectively, whereas the MBC values for MDR-PA and MRSA were 1.0 mg/mL and 2.0 mg/mL, respectively (Figure 2).

#### Biofilm Inhibition

**Figure 3:**
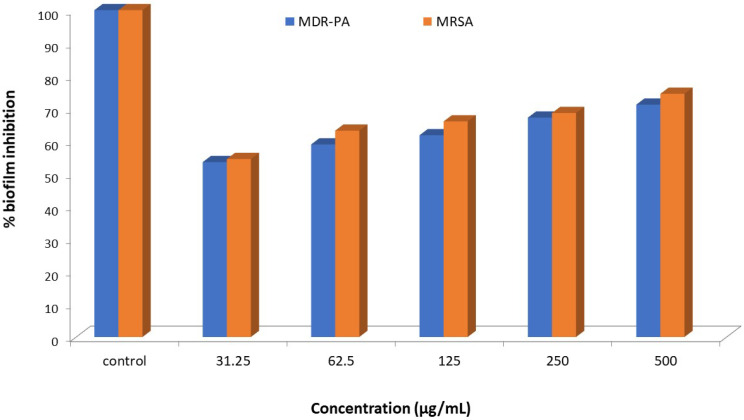
Effects of ZrO_2_/Ag_3_PO_4_ on biofilm-formation abilities of MDR-PA and MRSA at varying concentrations.

Figure 3 demonstrated the inhibition of biofilm formation of MDR-PA and MRSA by ZrO_2_/Ag_3_PO_4_. The MDR-PA cells treated with 31.25, 62.5, 125, 250, and 500 µg/mL of ZrO_2_/Ag_3_PO_4_ inhibited biofilm formation by 53.5, 58.9, 61.3, 67.1, and 71.1%, respectively. In comparison, under similar conditions, MRSA cells exhibited higher biofilm inhibition, i.e., 54.5, 63.1, 65.9, 68.5, 74.1%, respectively.

### 3.3. Structural and Degree of Conversion Analyses

**Figure 4:**
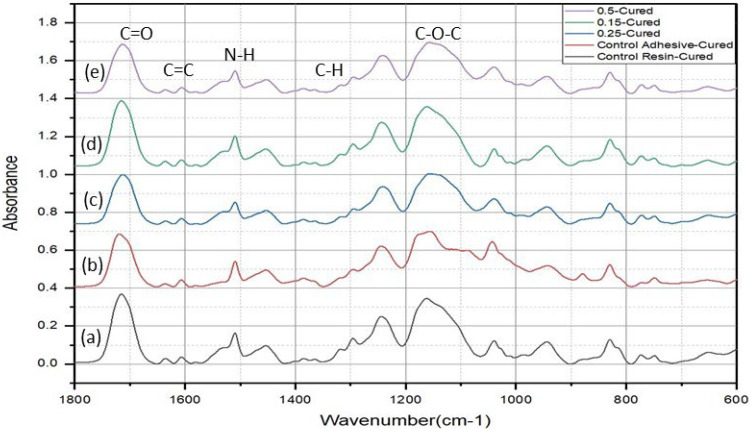
Comparative FTIR spectra of (**a**) Com, (**b**) Exp-0, (**c**) Exp-0.15, (**d**) Exp-0.25, and (**e**) Exp-0.5 after 10-min of curing. The characteristic peaks of C=O (1720 cm^−1^), C=C [aliphatic (1640 cm^−1^), aromatic (1611 cm^−1^)], bending N-H (1542 cm^−1^), bending C-H (1320 cm^−1^), and C-O-C (1245–930 cm^−1^).

The FTIR spectra (Figure 4) showed the characteristic peaks of cured dimethacrylate resin-based adhesives, and the assigned groups are mentioned in Table 1. The control adhesive spectrum showed a stretching O-H band at 3650–3000 cm^−1^, and the asymmetric and symmetric stretching peaks of C-H appeared at 2968 cm^−1^ and 2880 cm^−1^, respectively. The peak at 1720 cm^−1^ was attributed to C=O peak. The aliphatic and aromatic peaks appeared at 1640 cm^−1^ and 1611 cm^−1^, respectively. The peak at 1320 cm^−1^ corresponded to scissoring vibration of C-H presented in all of the constituent monomers. The peak at 1245 cm^−1^ was attributed to symmetric stretching of C-O in monomers. A weak peak at 930 cm^−1^ was attributed to the asymmetric stretching of C-O-C vibration. The experimental adhesives’ spectra exhibited almost same peak, except the stretching and bending N-H peaks appeared at 3310 cm^−1^ and 1542 cm^−1^, corresponding to the presence of urethane monomer.

**Figure 5:**
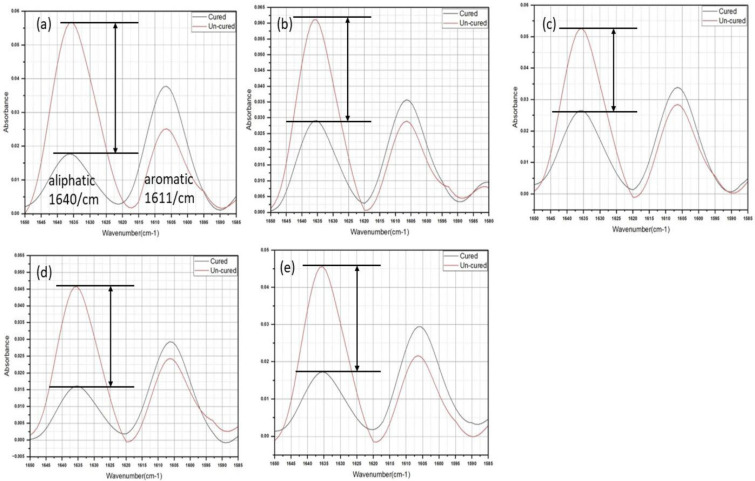
Comparative spectral peaks of uncured and cured (**a**) Com, (**b**) Exp-0, (**c**) Exp-0.15, (**d**) Exp-0.25, and (**e**) Exp-0.5 at the aliphatic (1640 cm^−1^) and aromatic (1611 cm^−1^) region. The decrease in peak height was observed in the aliphatic region mainly and degree of conversion was calculated for each group.

A band observed at the 777 cm^−1^ was attributed to C-N-H asymmetric stretching. It was found that with the increase in the concentration of the nanoparticles, the absorbance peak heights of carbonyl (1720 cm^−1^), aliphatic (1640 cm^−1^), and ester (1160 cm^−1^) were reduced. The comparative uncured and cured aliphatic/aromatic peaks are shown in Figure 5a–e. The reduction in aliphatic peak (1640 cm^−1^) after curing was observed for all the samples. The decrease in the peak height was due to the breakage of the covalent aliphatic double bonds C=C in the reacting monomers and formation of single covalent bonds C-C. The DC% obtained by the peak height and peak area analysis showed (Table 2) that the Com group showed the highest DC%, whereas among the experimental groups, the highest conversion was observed with Exp-0.25 and Exp-0.5 samples compared to other groups. However, a non-significant difference was observed between the Exp-0.25 and Exp-0.5, whereas a significant difference was observed with Exp-0 and Exp-0.15 groups.

### 3.4. Micro-Hardness Test

The Knoop hardness values are given in Table 1, and it was found that the Exp-0.5 group had the highest hardness value (39.11 ± 4.01) compared to all groups, whereby the Com had the lowest hardness value (17.21 ± 0.82). The statistical analysis showed that the *p*-value was significant between the groups (*p*-value = 0.000); thus, a pairwise comparison was made among the five groups using post hoc Tukey’s HSD test. The Exp-0.5 group showed a significant mean difference compared to all other groups (*p*-value = 0.000). Meanwhile, in Exp-0.25, a significant difference was found when compared to Exp-0.5 (*p*-value = 0.000), Com (*p*-value = 0.000), and Exp-0 (*p*-value = 0.001). The Exp-0.15 group showed a significant difference compared to Com (*p*-value = 0.000). The Exp-0 group, when compared to Com, had a significant difference (*p*-value = 0.000).

### 3.5. Flexural Strength and Modulus

The flexural strength and flexural modulus results are presented in Table 1 and a non-significant difference was found between the groups in both flexural strength and modulus. However, a significant difference was found between the control group and all experimental adhesives. Exp-0.25 and Exp-0.5 showed similar flexural strength and increased values compared to Exp-0.15 and Exp-0. However, among modulus data, the highest value was obtained for Exp-0.25.

### 3.6. Bond Strength Analysis

**Figure 6:**
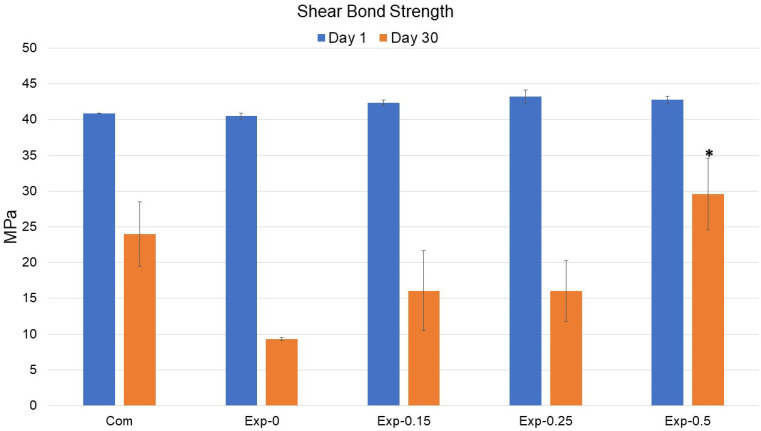
The graphic pattern of comparative shear bond strength of the control and experimental adhesives at days 1 and 30. At day 30, Exp-0.5 shows a significantly high value (labeled as *) compared to the other groups.

The shear bond strength test showed results at days 1 and 30 (Figure 6). At day 1, a non-significant difference was observed within the group where maximum bond strength was shown by Exp-0.25 (43.18 ± 0.94 MPa) and minimum by Exp-0 (40.47 ± 0.41 MPa). The values were significantly reduced at day 30 for all groups where Exp-0.5 showed statistically significant higher values (*p* < 0.5) followed by the Com. The Exp-0.15 and Exp-0.25 showed a non-significant difference in shear bond strength. All groups showed significant differences (*p* < 0.5) with Exp-0 group. It was found that compared to day 1, at day 30 the values of Com and Exp-0 groups showed a non-significant decrease in bond strength values. Exp-0.15 and Exp-0.25 showed a slight increase in value; however, the difference was non-significant. In contrast, Exp-0.5 showed an increase in values with a significant difference.

**Figure 7:**
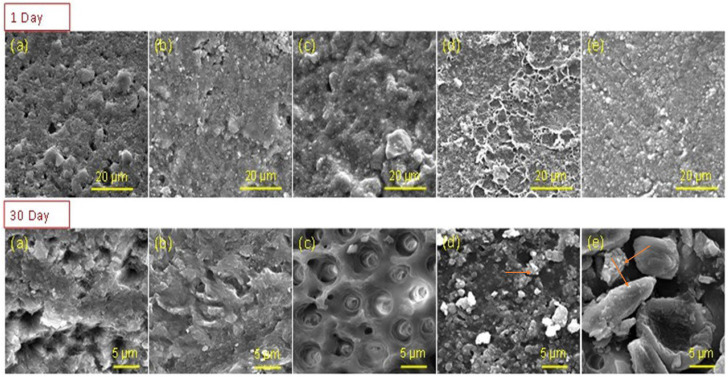
SEM images showing de-bonded surface of samples; (**a**) Com, (**b**) Exp-0, (**c**) Exp-0.15, (**d**) Exp-0.25, and (**e**) Exp-0.5. The de-bonded testing was done at days 1 and 30 of immersion in deionized water. The 30-days images showing presence of remnants of adhesives around dentinal tubules from groups Exp-0.25 and Exp-0.5, where the nanoparticles (arrows showing the presence of nanoparticles) are embedded in the resin matrix.

**Figure 8:**
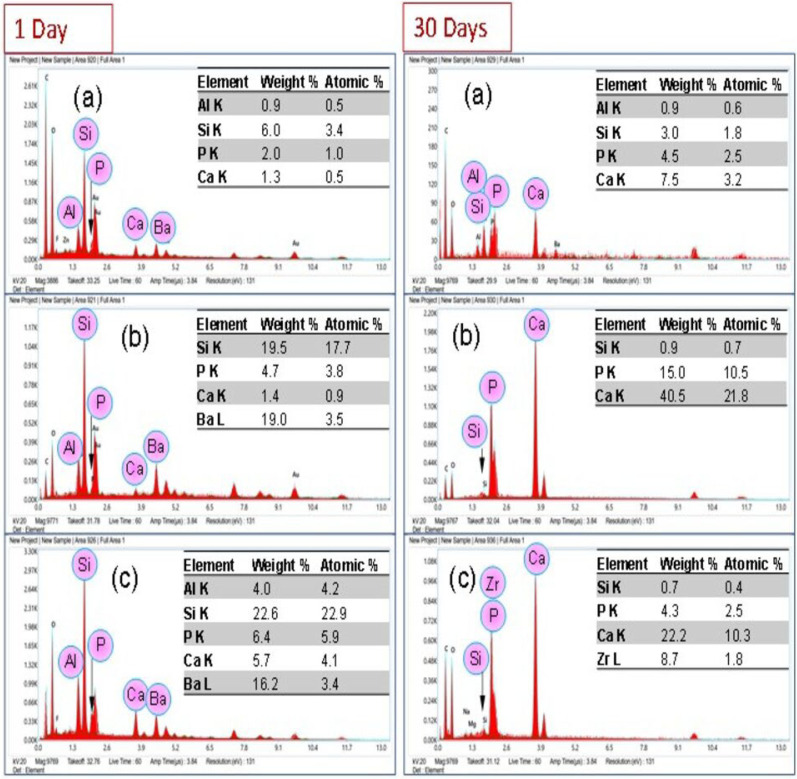
Elemental spectra and chemical composition of de-bonded surface of samples; (**a**) Com, (**b**) Exp-0, and (**c**) Exp-0.5. The de-bonded testing was done at days 1 and 30 of immersion in deionized water. The Au peak appeared due to the gold coating of the samples.

SEM images (Figure 7) of day 1 samples of each group showed remnants of resin on de-bonded surfaces. At day 30, the Com and Exp-0 showed the same behavior, whereby the dentin surface was covered with the remnants of adhesives after debonding. The Exp-0.15 sample after day 30 showed the appearance of dentinal tubules and a very thin layer of adhesives was present on the boundaries of the tubules. The tubules were partially occluded with the experimental adhesives. The images of Exp-0.25 and Exp-0.5 samples at day 30 showed presence of crystal-like structure along the dentinal tubule walls. These crystal-like structures could be the remnants of the experimental adhesives, as the nanoparticles are embedded (assigned with arrow in Figure 7d,e) within the matrix. The EDX spectrum (Figure 8) of Exp-0.5 confirmed the presence of Zr on the tooth surface, whereas the spectra of Com and Exp-0 showed mainly Ca and P (from tooth surface). The ARI showed that at day 1, the average score for all groups was 3. For Exp-0, half of the samples showed a score 2, and the rest showed 3. At day 30, the average score for Exp-0 was 4, whereas, for other groups, the average scores were 3 with a non-significant difference.

### 3.7. Color Change Measurement

The color change (ΔE) data is tabulated in Table 3. Up to day 30, more color shift was found with Com, Exp-0, and Exp-0.15 groups compared to Exp-0.25 and Exp-0.5. Exp-0.25 and Exp-0.5 showed significant differences compared to the other groups, whereby a non-significant difference was found among other groups. However, day 1 vs. day 60 results showed that the ΔE values were increased for Com, Exp-0.25, and Exp-0.5 groups, whereas no difference was found for Exp-0 and Exp-0.15 groups. A non-significant difference was found within the groups.

## 4. Discussion

The introduction of nanoparticles has shown its impact on dental restorative materials whereby recently, smart nanoparticles have received much interest for their uses in dental applications [36]. Dental adhesives based on nanomaterials with bioactive, antibacterial, and biocompatible properties have been studied; however, very few of them were marketed and became available for clinical applications [37,38]. It is claimed that the inclusion of nanomaterials in dental adhesives can reduce bacterial viability [39], improve mechanical properties and interfacial bonding [40], reduce polymerization shrinkage and water sorption, and inhibit enzymatic and chemical degradation [41,42,43,44,45].

In this study, to overcome the conventional drawback of dental adhesives and to improve the properties, phosphate-based nanomaterials containing silver (Ag), zirconia (ZrO_2_), and phosphate (PO_4_) were synthesized and incorporated in dental resin matrix. This study showed that the change in physical and mechanical properties was observed with the incorporation of nanomaterials. The physical property (micro-hardness) and mechanical properties, i.e., flexural strength/modulus and bond strength, were improved with the increased concentration of nanoparticles.

The nano-structure of the particles was confirmed with TEM analysis, and phase purity was analyzed with the XRD. DRS have been used as a standard technique for measuring the absorption and to determine the optical band gap. It is reported [46] that the band gap can be altered with ion substitutions. A similar trend was observed in this study, where the nanoparticles showed absorption at visible range. The absorption was observed at 245 nm and 450 nm. The Ag_3_PO_4_ excites by radiation in UV-vis range and as a secondary effect, there is a formation of silver nanoparticles in situ by the reduction of Ag^+^ from Ag_3_PO_4_. The results are incoherent with the previous study [47], where ion doped zirconia was studied and a strong band appeared at 470 nm and was attributed to electrons trapped at oxygen vacancies nearest to the zirconium cations. Another study [48] showed the presence of an absorption band of the monoclinic zirconium near 290 nm and increased absorption (400–700 nm) could be attributed to the presence of defect states. It is established that these defects have a significant effect on the optical and luminescent properties [49]. In comparison to these studies, the reduced absorption observed in the current study could be due to the presence of silver and the change in internal structure [50].

The main purpose of utilizing these synthesized nanoparticles is to obtain their antibacterial properties. In this study, the MIC and MBC data clearly showed that ZrO_2_/Ag_3_PO_4_ was more effective against Gram-negative bacteria than Gram-positive bacteria. This might be due to structural differences in the cell walls of both types of bacteria. It is well known that the major constituent of the cell wall of Gram-positive bacteria is peptidoglycan (20–80 nm) layers which are thick and rigid, providing additional protection, whereas the cell wall of Gram-negative bacteria contains a thin layer of peptidoglycan (7–8 nm); however, it also has a highly negatively charged lipopolysaccharides layer.

In this study, Tetric N-bond Universal was used as control group. This commercial adhesive contains methacrylate-based resin and silica as filler, and ethanol is used as a solvent. It was maintained in this study that the experimental adhesive should also be based on methacrylate-based resins, fillers, and solvent. The silanized nanoparticles were successfully incorporated into dimethacrylate resins. The uniform distribution can be justified by the mixing method adopted in this study. It was reported previously that mixing followed by ultrasonication formed stable suspensions and decreased sedimentation during storage [51]. During the optimization process in this current study, it was observed that the nanoparticles were not sedimented. Furthermore, the nanoparticles might be better distributed/dispersed in the resin matrix. The silanization of the fillers is an important factor to obtain better linkage with the resin matrix, subsequently affecting the physical and mechanical properties. The combination of bis-GMA, TEGDMA, and UDMA was used to prepare dental adhesives. Hydroxyethyl methacrylate (HEMA) was not used in this composition due to its high hydrophilic properties. The presence of hydrophilic monomer may increase water diffusion into the adhesive layer after polymerization, leading to hydrolytic degradation and thus lower durability [52]. It is also reported that HEMA has negative effects on the mechanical properties, bond strengths, and polymerization of the adhesives [53]. Further, HEMA can release from adhesives and move towards pulp via dentinal tubules, subsequently causing cytotoxicity and genotoxicity [54]. Therefore, HEMA was replaced with UDMA to reduce the aforementioned drawbacks. UDMA has been frequently used as a bonding agent due to better flexibility, low solubility, low water-absorbing characteristics, and its ability to inhibit transesterification [55]. The comparative spectra of the experimental adhesives confirmed the presence of urethane with stretching and bending peaks of N-H at 3310 cm^−1^ and 1542 cm^−1^, respectively. These peaks did not appear in the commercial adhesive due to the absence of UDMA in this adhesive. In this study, 1611 cm^−1^ peak was used as an internal standard, and 1716 cm^−1^ (carbonyl) was not used; however, previously it had been used as an internal standard [56]. The FTIR results of the present study showed changes not only in the aliphatic peak, but changes in peak height were also observed at the carbonyl peak after curing. It is reported that the vibrations of vinyl and carbonyl groups are not independent and that their conjugation is lost on polymerization [57,58]. Therefore, the carbonyl peak was not used as an internal standard.

The DC% was evaluated with FTIR, and spectral readings were taken immediately after curing, and then after 10 min. Our previous study reported that the reaction kinetics of resin-based restorative materials attained the maximum polymerization after 24 h [59]. A similar trend was observed in this study, where spectral changes were observed after immediate curing and after 10 min. The change in band/peak height and width demonstrated that in the initial phase of polymerization process, the presence of nanoparticles did not hinder the polymerization process. The improved mechanical properties, reduced polymerization shrinkage, and reduced water sorption are dependent upon the cross-linking density and structural quality of the network formed during polymerization [60]. Higher DC% can lead to superior mechanical properties of the resin adhesive [61]. The experimental adhesives had a promising role in the adhesive system, expressed by the DC% test. The DC% of the Exp-0.25 and Exp-0.5 was higher than the Exp-0. This reflects the significant role of nanoparticles in dental adhesives. The success and stability of adhesives depend on the quality and quantity of fillers [60]. The presence of fillers in weight concentration and their surface chemistry, shape, size, uniform distribution, presence of hydrophobic resins, and filler–resin interaction determine the adhesive longevity [62]. The addition of fillers can increase the viscosity of the adhesives, which can inversely effect the degree of conversion. However, in this study, with the increase in the concentration of fillers, the degree of conversion was not reduced but rather increased. The concentration (0.15–0.5 wt.%) did not alter the viscosity and did not negatively affect the degree of conversion. This is in agreement with a previous study [63]. It is important to consider the concentration of the fillers in dental adhesives. During the optimization process, it was found that the fillers with 1 wt.% more negatively affect the degree of conversion. Therefore, 0.5 wt.% was considered as the maximum load in the resin matrices. In this study, the nanoparticles were silanized to get better interaction with the resin matrix, whereby silanization contributed to immobilize fillers in the resin matrix. Previously, our group showed that silanization of nanoparticles had a positive effect on the physical and mechanical properties of resin-based materials [64]. The experimental groups in this present study had lower values than the commercial group; however, all groups exhibited an acceptable range of conversion (52–75%) [65].

Micro-hardness is one of the physical properties that can be increased by adding fillers to the adhesive system [66]. In accordance with these, the outcome of the present study showed a statistically significant increase in micro-hardness, specifically in the experimental group with the higher nanofiller content. The Exp-0.5 group showed statistically significant results when compared to all other experimental groups and the commercial reference. The flexural strength data also showed the same behavior, where experimental adhesives showed high values compared to the control group. It is assumed that the increased mechanical values could be in correlation to increased DC% and uniform distribution of the nanoparticles. The micro-hardness provides the surface property and flexural strength provides the inner strength of the material. The other reason for high values could be due to an increased concentration of nanoparticles and the increase in percentage of hard phase of nanoparticles inside the ductile resin matrix system [67].

The adhesive–dentin interfacial linkage and bond strength tests have been used previously to evaluate the performance of dental adhesives to obtain the best possible material for clinical applications [68,69]. Therefore, it is important that new experimental adhesive systems should be carefully evaluated before proceeding toward clinical usage. Over the period, many tests have been used to evaluate the adhesiveness; however, no methodology can simulate the exact clinical variables. The micro-tensile and shear bond testing are the two most commonly used techniques, each with their advantages and disadvantages. Heintze et al. [70] reported that the literature lacks consensus on the acceptance for each bond strength test, and the adhesiveness should be compared before and after aging to obtain a correlation between the results and clinical acceptance parameters. The shear bond test is still the most commonly used due to the ease of specimen preparation, simple testing protocol, and lower incidence of pretest failure [71].

To the best of the authors’ knowledge, this is the first study where Ag/ZrO_2_/PO_4_ nanoparticles were incorporated into the adhesive resin. The experimental adhesives showed favorable bond strength values at day 1 and with aging for 30 days. The SBS test demonstrated linear behavior such that with an increase in the concentration of the nanoparticles, the values were increased significantly compared to the unfilled resins. The results of the present study are in agreement with previous studies, which showed that with the addition of nanofillers, the bond strength to dentin increased [72,73]. It is expected that the presence of silver phosphate contributed to the apatite formation, and a difference in calcium/phosphate ratio was observed. The new apatite formation can eventually improve the strength and linkage with the restoration. The failure pattern was analyzed by ARI scoring, and it is an important parameter to determine the amount of adhesive remaining on the dentin surface. The greater amount of adhesive on the surface is related to the higher bond strength values and exhibits cohesive behavior. In this study, mostly mixed behavior was observed, with no statistical difference in ARI scoring within the groups. Many factors can influence the bond strength and adhesive/cohesive behavior, including the substrate type, substrate deepness and location, dentinal tubule direction, tooth extraction time, and storage [74]. In this study, the extracted teeth were stored in a thymol solution, and it is reported that storage of teeth in a thymol solution for up to 6 months could not influence the shear bond strength [75].

In this study, higher loading was performed before finalizing the ratio of nanofillers in adhesives; however, the adhesives showed a dark appearance, and a low DC% was achieved. Therefore, a maximum of 0.5 wt.% was optimized as a reinforcing agent. Considering the weight percentage of nanofillers in a resin matrix is important. Another important aspect to consider is the difference in refractive index of the fillers and resin. The nanofillers have a tendency to agglomerate, subsequently causing poor penetration through the inter-fibrillar spaces and the appearance of voids within the hybrid layer [76]. The SEM images showed adhesive and mixed behavior mainly and no droplets were observed, indicating no entrapment of solvent in adhesive layer. In this study, ethanol was used as a solvent, which could help in the interaction between monomers and dentinal water. It is reported that 10–20% of residual ethanol is useful to improve the degree of conversion; however, this can reduce the physical and mechanical properties [77,78]. The results of the control group and unfilled adhesive after 30 days are in accordance with previous studies where with time, the values decreased. The decreased values could be due to hydrolytic degradation, whereby the water storage can cause swelling and plasticization, consequently decreasing the mechanical properties [79,80]. In contrast, the experimental groups with higher concentration of nanoparticles showed improved values. This could be due to the affinity of nanoparticles with the resin matrix and providing a more hydrophobic bonded interface [81]. It is anticipated that the hydrophobicity was increased due to changes in surface energy of the dentin substrate resulting in better wettability of demineralized dentin surface leading to enhanced infiltration of adhesive monomers [82]. The SEM images showed encapsulation of collagen fibrils with adhesives, which could contribute to improving bond strength and durability. The images showed that the nanoparticles were present within the resin matrix and it is expected that the reinforcement would increase the strength of the adhesives. Furthermore, the ion leaching would improve the antibacterial activity.

In this study, the nanoparticles were based on silver, and it was anticipated that the silver could influence the color of adhesives, and subsequently, the composite restoration. It has been reported that adhesive materials could interfere with the optical properties of the composite restorations. Therefore, the color stability of the composite restoration was investigated after applying the adhesives underneath. More color change (ΔE) after 60 days was observed with the commercial adhesive; however, the non-significant difference was observed with Exp-0.25 and Exp-0.5 groups. The acceptable ΔE for CIELab is 2.7, and it is considered unacceptable if it exceeds 3.3 [83,84]. In this study, after 60 days of immersion in deionized water, the values were almost in an acceptable range, whereby the ΔE for commercial adhesive and Exp-0.5 were slightly high, i.e., 2.97 and 2.83, respectively. The color change in the control and experimental adhesives was related to the difference in the composition, reaction kinetics, and degree of conversion [85]. The presence of camphorquinone and tertiary amine could also contribute to color changes [86].

The presence of silver in nanoparticles is one factor which can interfere with the color stability, however, it was co-synthesized with zirconia and phosphate. Previously, it has been reported that the addition of 0.2 wt.% of silver nanoparticles in adhesives reduced the resistance and changed the color of the materials [87]. In this study, the maximum concentration of nanoparticles was 0.5 wt.%. The rationale for using a silver-based nanoparticle was to obtain the benefit of its antibacterial characteristics. A previous study reported that 0.005–0.025 wt.% of pure silver particles was considered 250 ppm, an optimal concentration for bacteriostatic and bactericidal effects [88]. This study must further investigate the antibacterial characteristics of these experimental adhesives. Further, the cytocompatibility of these experimental adhesives should be investigated in detail.

## 5. Conclusions

Within the limitations of this study, it is concluded that the addition of ZrO_2_/Ag_3_PO_4_ nanoparticles to the resin matrix yielded significant improvement in the physical and mechanical properties of the experimental dental adhesive. TEM analysis revealed the nano-structure, and the nanoparticles showed antibacterial activities against both the Gram-positive and Gram-negative bacteria. Structural analysis showed that the degree of conversion was affected by the concentration of nanoparticles; however, the percentage was in a clinically acceptable range. The flexural strength and bond strength of Exp-0.5 showed better results compared to other groups. The mixed adhesive/cohesive behavior was observed on the dentin surface. Long-term color stability was achieved for all samples. Overall, Exp-0.5 showed significantly superior outcomes compared to the other groups. The combination of ZrO_2_/Ag_3_PO_4_ and dental resin matrix has not been used before and formulation of these produced the experimental adhesives. The studied physical and mechanical properties provided a base to use these materials for dental application. Based on these findings, it is recommended that the experimental adhesives have potential to be used for clinical applications; however, further biological studies such as those on cytotoxicity and antibacterial activity should be performed in future.

## Figures and Tables

**Table 1:** The observed peaks and assigned groups after curing of the dimethacrylate-based resins.

Wavenumber (cm^−1^)	Assignned	Reference
3650–3000	Stretching O-H	[32]
3310	Stretching N-H	[33]
2968	Asymmetric stretching C-H	[34]
2880	Symmetric stretching C-H	[33,35]
1720	C=O	[34]
1640	C=C Aliphatic	[33,35]
1611	C-C Aromatic	[33,35]
1542	Bending N-H	[35]
1320	Bending C-H	[35]
1245	Symmetric stretching C-O	[34]
930	Asymmetric stretching C-O-C	[34]

**Table 2:** The mean (SD) DC (%), micro-hardness, flexural strength, and modulus of the control and experimental dental adhesives.

	DC (%) Peak Height/Peak Area	Micro-Hardness (KHN)	Flexural Strength (MPa)	Flexural Modulus (GPa)
**Com**	73.68 ^a^ (2.14)/76.46 (2.54)	17.21 ^a^ (0.82)	105.58 ^a^ (4.70)	1.40 ^a^ (0.19)
**Exp-0**	55.73 ^a^ (1.50)/56.32 (2.05)	24.30 (1.30)	174 (10.66)	2.74 (0.62)
**Exp-0.15**	54.71 ^a^ (2.50)/56.15 (2.90)	27.35 (2.24)	184.37 (7.59)	3.26 (0.16)
**Exp-0.25**	66.66 (1.80)/70.76 (2.50)	30.50 (2.30)	188.55 (7.20)	4.24 (0.46)
**Exp-0.5**	64.44 (3.65)/69.04 (3.05)	39.11 ^a^ (4.01)	187 (8.51)	3.45 (0.40)

^a^ significant difference (*p* < 0.5).

**Table 3:** Color change (ΔE) showing a comparison of day 1 vs. day 30 and day 1 vs. day 60 of the control and experimental dental adhesives.

	Com	Exp-0	Exp-0.15	Exp-0.25	Exp-0.5
Day 1 vs. Day 30	3.27 (0.44)	2.66 (0.63)	2.70 (2.25)	1.33 ^a^ (0.40)	1.06 ^a^ (0.09)
Day 1 vs. Day 60	2.97 (0.82)	2.58 (0.01)	1.833 (0.42)	2.16 (0.94)	2.83 ^b^ (1.07)

^a^ statistically significant difference. ^b^ statistically non-significant difference

## Data Availability

Not applicable.
